# Identifying and Classifying Trait Linked Polymorphisms in Non-Reference Species by Walking Coloured de Bruijn Graphs

**DOI:** 10.1371/journal.pone.0060058

**Published:** 2013-03-25

**Authors:** Richard M. Leggett, Ricardo H. Ramirez-Gonzalez, Walter Verweij, Cintia G. Kawashima, Zamin Iqbal, Jonathan D. G. Jones, Mario Caccamo, Daniel MacLean

**Affiliations:** 1 The Sainsbury Laboratory, Norwich Research Park, Colney, Norwich, United Kingdom; 2 The Genome Analysis Centre, Norwich Research Park, Colney, Norwich, United Kingdom; 3 Wellcome Trust Centre for Human Genetics, University of Oxford, Oxford, United Kingdom; Harvard Medical School, United States of America

## Abstract

Single Nucleotide Polymorphisms are invaluable markers for tracing the genetic basis of inheritable traits and the ability to create marker libraries quickly is vital for timely identification of target genes. Next-generation sequencing makes it possible to sample a genome rapidly, but polymorphism detection relies on having a reference genome to which reads can be aligned and variants detected. We present Bubbleparse, a method for detecting variants directly from next-generation reads without a reference sequence. Bubbleparse uses the de Bruijn graph implementation in the Cortex framework as a basis and allows the user to identify bubbles in these graphs that represent polymorphisms, quickly, easily and sensitively. We show that the Bubbleparse algorithm is sensitive and can detect many polymorphisms quickly and that it performs well when compared with polymorphism detection methods based on alignment to a reference in *Arabidopsis thaliana*. We show that the heuristic can be used to maximise the number of true polymorphisms returned, and with a proof-of-principle experiment show that Bubbleparse is very effective on data from unsequenced wild relatives of potato and enabled us to identify disease resistance linked genes quickly and easily.

## Introduction

Genetic polymorphisms between genomes of individuals in a population, such as Single Nucleotide Polymorphisms (SNPs), are invaluable markers for tracing the genetic basis of inheritable traits or diseases. Rapid detection of polymorphisms and creation of large libraries of SNPs is vital for timely investigation and identification of genes associated with medically and agronomically important phenotypes. Next-generation sequencing (NGS) can sample genomes comprehensively in only hours, but making use of the typically short reads remains a challenge. Detection of SNPs and short Indels is typically achieved by aligning reads to a reference genome and identifying where the consensus from the aligned reads differs from the reference sequence. Factors such as the need for a reference sequence and the implicit assumption of a monomorphic sample mean that the consensus approach is limited in organisms for which we lack a reference genome, outbred diploid samples, bulked population data or analysis of metagenomes. The ability to identify genetic differences between two (or more) sequenced but not assembled, and genetically divergent genomes would be of great benefit to biotechnologists wishing to create libraries of genetic markers for breeding and disease programs in as short a time as possible. The major obstacles to identifying genetic differences directly from NGS reads are the volume of sequence data that must be assessed and the accurate detection of variants in reads that individually are often prone to error. But even with perfect reads the alignment-based methods will struggle with complex genomic regions or in the presence of indels.

**Figure 1:**
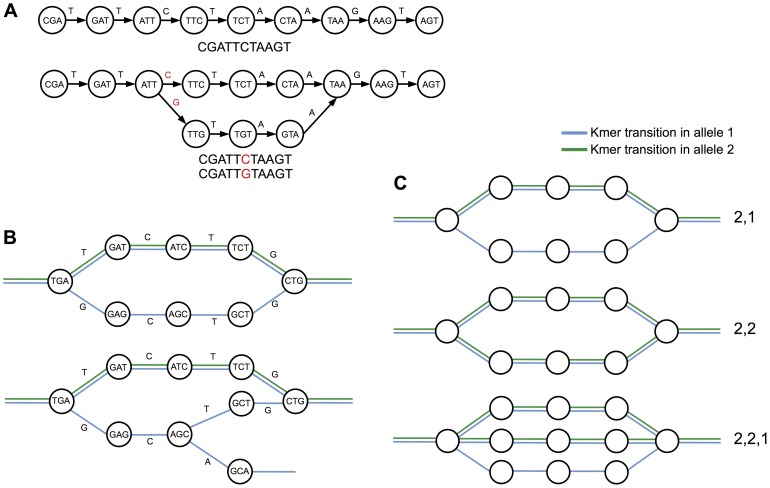
Bubbles in the de Bruijn graph. (A) Representation of a simple 11 nt sequence as a de Bruijn graph (top) and then with a SNP (bottom). Nodes represent *k*mers – sequences of *k* nucleotides – and edges join together kmers that overlap by *k*−1 nucleotides. A SNP causes a bifurcation in the graph and the new path joins up with the original path after *k* nodes. (B) de Bruijn graph representations of a single heterozygous SNP (top) and a SNP followed by a second SNP within *k* nt (bottom). (C) Our bubble classification system assigns a type according to the number of colours present on each path through the bubble. Thus a bubble corresponding to a heterozygous SNP from an organism in which the resistant variant contains 2 alleles and the susceptible contains 1 allele would produce a bubble with 2 colours on one path and 1 colour on a second path and would be classified as a type “2,1”. Similarly, a bubble corresponding to a heterozygous SNP from an organism in which both the resistant and susceptible variants contain 2 alleles would be classified as a type “2,1”. Finally, a less common example where 2 alleles are present in one variant and 3 in another would appear as a type “2,2,1”.

De Bruijn graphs are directed graphs of overlapping symbols that are directly suited to representing ordinal relationships between same length sub-sequences of reads (typically called *k*mers, being of arbitrary length *k*) (Figure 1A). They can be implemented in efficient data structures for large collections of kmers and have proven to be of great utility as the underlying data model over which numerous de novo assembly algorithms have been implemented [1], [2], [3].

Methods based on the de Bruijn graph have been implemented recently that can efficiently identify differences in sequence read sets [4] but do not yet extend to allowing direct classifications based on genetic background. Some very specialised pipelines have also been developed for the tracking of genetic loci through populations on reduced representation Illumina data [5]. Iqbal *et al.* [6] produced the first model and de novo assembly algorithms for variant discovery and genotyping from sequence data without using a reference genome. By encoding different datasets in different colours in the graph (e.g. each colour representing a single sample, or a single pool/population), they introduced novel methods for detecting variants and distinguishing populations or individuals by finding alleles with differential colouring. They described how variants induce “bubble” structures in the graph, which could be confounded with sequencing errors or repeated kmers. Given a specific genome of interest, a variant-size and a kmer size, the power to detect variants was modelled. The authors noted that without a reference, it was difficult to distinguish read errors and repeats from genuine SNPs.

We present here an extension of the method in Iqbal et al. [6], which has in principle greater power to discover SNPs. The method relies on implementing a minimal error-cleaning routine, followed by a depth-first search in the graph to find bubbles and has the explicit goal of reliably identifying real and useful homozygous and heterozygous SNPs from samples of varied genetic background. The method relies on differential coverage on the different branches of a bubble from two different samples. For example a homozygous SNP in a resistant individual or population would appear in the graph as a bubble with two different coloured branches, that is kmers from only one sample on each side of the bubble. Similarly a heterozygous allele in a disease resistant organism that is homozygous in a susceptible variant would manifest as a bubble with a dual coloured branch for the allele for susceptibility (Figure 1C). We control specificity by ranking called variants using differential coverage on the two alleles. We demonstrate the power of combining multicoloured graphs with an appropriate experimental design, by identifying SNPs linked to *Phytophthora infestans* resistance in crosses of resistant and susceptible accessions that are relatives of potato.

## Results and Discussion

Our software for identifying SNPs, Bubbleparse, uses the efficient de Bruijn graph representation provided by Cortex [6], and implements a new algorithm for identifying bubbles. Though Cortex provides a method of variant calling, the algorithm is designed only to detect clean bubbles and more complicated structures require a reference for identification. Our algorithm identifies more complex bubbles without the use of a reference. Given sufficient coverage, the method can call all possible variants, but also errors, paralogs and misassemblies. Therefore we control specificity with a classification and ranking system that we benchmark below and show to be effective.

### Bubble detection

A de Bruijn graph is a labeled directed graph represented by a pair 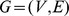, consisting of a set of vertices, 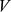, and a set of edges, 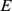. Each vertex (or node) of the graph represents a *k*mer, which is a nucleotide sequence of length *k* that differs from connected *k*mers by a single nucleotide.

A path through the graph can be represented by 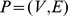, where 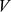 is a set of 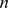 vertices, 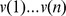 and 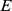 is a set of 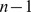 edges, 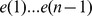, such that the target node of 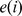 is the source node for 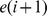.

Two paths, 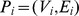 consisting of 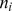 vertices and 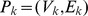 consisting of 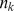 vertices, form a bubble if
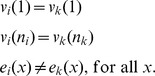



In the first step of bubble finding, we examine each node in the graph looking for one of two kinds of possible branch point.: ‘Y’ nodes feature one path in one direction and 2 (typically) or more (up to 4, one for each nucleotide) in the opposite direction; and ‘X’ nodes feature 2 or more paths in both directions. Branch points are places where the path through the graph diverges and if these paths later re-converge, a bubble is formed. Therefore, the next step is to explore each node flagged in the first step and see if the paths originating from it later converge on a common node. This is achieved by building a list of all paths from a given branch node, up to a maximum number of bifurcations traversed, which is a depth-first search. These paths are then compared step-by-step, looking for a common node of convergence. A parameter, *d*, determines the number of levels of bifurcation that will be allowed when exploring potential bubble paths. A value of 1 means that 1 level of bifurcation will be explored before the algorithm abandons further exploration of the path. Thus, the maximum number of paths that will be explored is equal to 4^1+d^.

When a point of convergence is identified, we have the start and end nodes of a bubble and can easily produce a set of nucleotide sequences representing the paths through the bubble. For practical use, such as the design of primers, we need to find flanking sequences at either side of the bubble. These are obtained by walking the graph as far as possible from the start and end nodes, until we reach another branch node.

This bubble detection algorithm has been implemented as a new module within Cortex, independent of and separate from the standard variant calling options. The primary advantage of our new algorithm is that it allows the reference-free detection of more complicated bubble structures than Cortex does (see below for experimental verification), but also that it allows us control over the information that is available to the Bubbleparse ranking algorithm. The output of this new module is a pair of files that can be read by the Bubbleparse ranking tool. Once the collection of bubbles is created, Bubbleparse gives each a type classification determined by the number of paths through the bubble and the number of colours that follow each path (Figure 1C). Bubbleparse creates these classifications and collates quantities such as node coverage per colour path, kmer quality score, coverage ratio and produces a ranked list of SNPs subdivided according to bubble type.

### Bubble detection and classification is sensitive and fast but creates many false positive SNP calls at greater graph search depths

The reference free exploration of the graph is expected to generate high numbers of false positives induced by repeats in the underlying genome and numerous artefacts in the sequence reads that can cause bubble structures that are not due to polymorphisms. In order to quantify this error level and to examine the efficiency with which we could detect true SNPs in bubble collections from experimentally generated reads, we compared our SNP calls with those from a commonly used SNP calling pipeline based on reference alignment, as well as with a curated high-quality set of SNPs from an external genetic variation project. We used reads from the Bur-0, Tsu-1 (31–40 nt) and Ler-1 (40–80 nt) ecotypes of *Arabidopsis thaliana* released by the 1001 Genomes Project and available from the Sequence Read Archive at EMBL (ENA SRA Experiments SRX000702 to SRX000704), against *A. thaliana* Col-0 reads. The Bur-0 reads provided approximately 50× coverage, the Tsu-1 reads 38× coverage, while for Ler-1 we present results for a range of coverage values from 40× to 340×.

Alignment against the reference was performed using BWA [7] and SNPs called using SAMTools [8]. In order to have a high degree of confidence that a homozygous SNP was genuine, we required 95% of the reads covering a position to carry the same nucleotide and for that nucleotide to be different to the one in the same position in the Col-0 reference. For Bur-0, this resulted in SAMTools calling 351,493 and 182,419 SNPs at minimum alignment coverage of 10 and 20 respectively. For Tsu-1, the values were 261,970 and 88,597.

In Bubbleparse, reads were assembled into the de Bruijn graph, removing paths of nodes with coverage 2 or below and tips less than 100 nucleotides in length. Bubble detection was run using depth from 0 to 3 and k from 15–31. The contigs representing bubbles were BLASTed [9] against the TAIR9 reference in order to find their locations and to build a list of predicted SNP locations.

**Figure 2:**
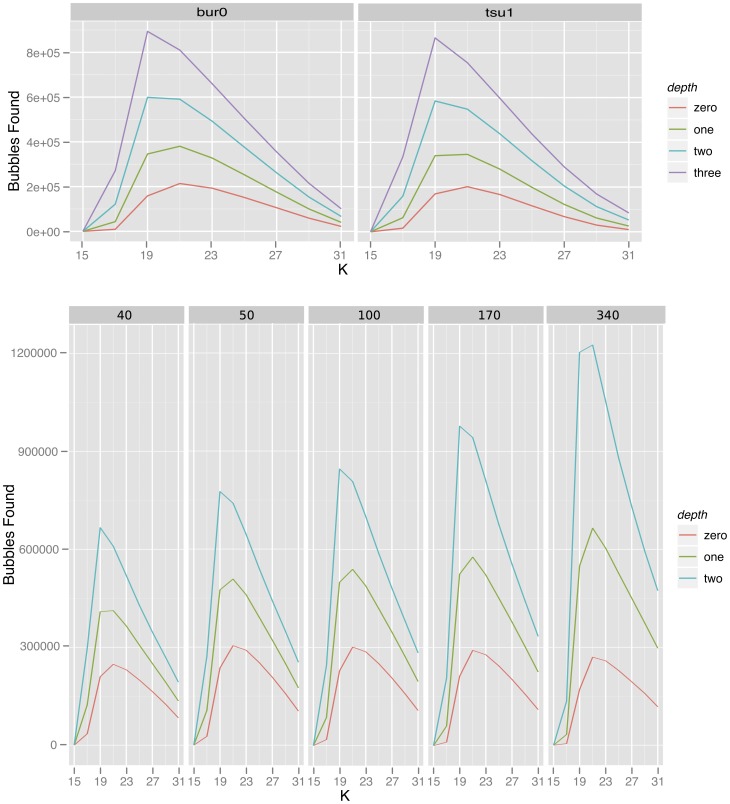
Effect of depth of search on number of bubbles found by Bubbleparse. Graphs showing numbers of bubbles found for Bur-0 and Tsu-1 with search depth set to 0, 1, 2 and 3 at constant read coverage (top) and for Ler-1 at varied read coverage (bottom).

Bubbleparse identified many bubbles in each comparison (Figure 2), (maximum 893,627 for Col-0/Bur-0 at depth 3 and k = 19. The number of bubbles found increased with depth of search as expected and roughly mirrored the number of distinct kmers, which were maximal at k = 19 or k = 21 for all Bur-0 and Tsu-1 (Figure S1), but decreased sharply as k moved away from this.

**Figure 3:**
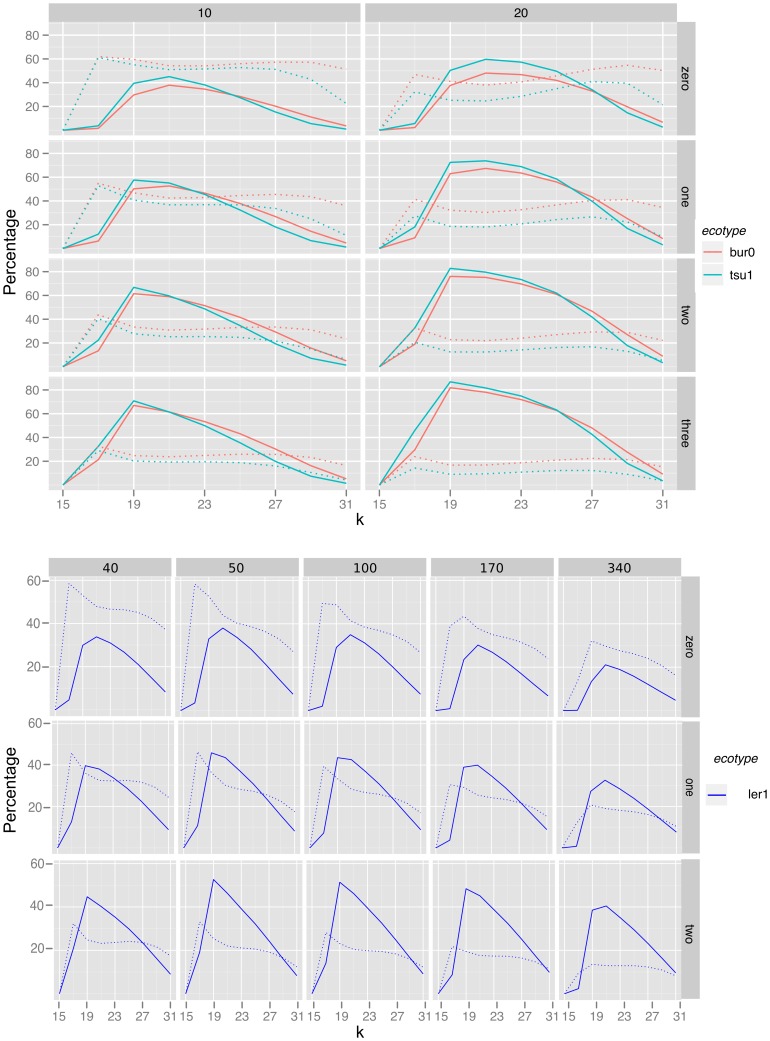
Identification of *Arabidopsis thaliana* SNPs. Percentages of canonical SNPs found (solid lines) and percentage of Bubbleparse identified SNPs that were found in the canonical set (dotted lines) for Bur-0 and Tsu-1 with search depth set to 0, 1, 2 and 3 at constant read coverage (top) and for Ler-1 at varied read coverage (bottom).

Comparing the canonical SNP positions, obtained by pileup, with the Bubbleparse matches allowed us to estimate sensitivity and compare this with the proportion of real SNPs retained in our bubble collections. The number of real SNPs found in our bubble collections increased with depth of search to a maximum of 86.82% of SNPs identified in Tsu-1 (Figure 3) for depth 3 indicating that the search algorithm is effective at detecting bubbles representing real SNPs. Figure 3 shows that this sensitivity comes at the cost of a significant amount of specificity at greater search depths, such that the proportion of real SNPs in the collection drops from 61.99% at its highest (for Bur-0 at k = 17 and bubble search depth of 0 with minimum coverage for calling a canonical SNP of 10) to 3.53% at its lowest (for Tsu-1 at a bubble search depth of 3 and minimum read depth coverage for calling a canonical SNP of 20 at k = 31). Together these results indicate that the graph traversal algorithm is effective at finding bubbles and that the simple bubbles discovered by low depth graph searches are more likely to represent a SNP than the complex structures discovered by a deeper graph search. We believe that the complex structures in the graph may more often represent underlying genomic characteristics such as k length repeats or read errors, rather than differences in sequence between ecotypes.

### Too much read depth can reduce rates of SNP detection and produce higher rates of false positives

To test the effects of high read coverage and to compare our data with a heavily examined and well-curated canonical set of SNPs we carried out a similar analysis with the *Arabidopsis* ecotype Ler-1. For this, we used the 1001 Genomes projects own list of SNPs, generated as described by Schneeberger *et al.* [10]. We considered these lists of SNP positions as a reference set against which to compare those discovered by our method. For Ler-1 we varied the average coverage of kmers in reads sent to Bubbleparse rather than varying the depth coverage limit for the reference set SNP calling as for Bur-0 and Tsu-1, up to the maximum available 340× (Methods S2). We observed a similar pattern to that with the previous ecotypes, many bubbles were found to a maximum 1,226,068 at depth 2 for k = 21, and sensitivity was of similar magnitude with a maximum of 52.66% of canonical SNPs found at optimum k for bubble counts as in the Bur-0 and Tsu-1 data (Figure 2B). The efficiency of the search for real SNPs is best close to the k value that maximises the kmer count. Also as before, increasing the depth of search into the graph increases the sensitivity at the cost of false positives. Increasing the depth of sequencing coverage did little to improve the sensitivity or number of false positives with modest gains from 40× to 50× and decreases after that (Figure 3B). While a basic level of coverage is required to give confidence that bubbles are the result of SNPs and indels in the target organism, increasing the number of reads also increases the amount of error in the data, causing the formation of false bubbles and increasing the complexity of bubbles formed by polymorphisms. The similarity in pattern of results between the ecotypes indicates that the algorithm is robust to differences in input data and behaves reproducibly between sets.

### Bubble search and classification is fast

Table S1 provides demonstrative execution times for assembly of de Bruijn graph, identification of potential SNPs and output of contigs for the Col-0 vs. Tsu-1 experiment. Assembly and classification were carried out on a range of individual machines within a workload managed cluster containing nodes with up to 128 Gb RAM available. The maximum sensitivity searches are complete with mean time 29 h.

### Comparisons with existing tools

**Figure 4:**
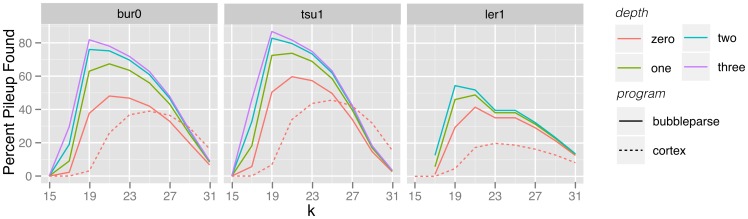
SNP finding in Cortex_var and Bubbleparse. Percentages of canonical SNPs found by Cortex_var (dotted lines) and by Bubbleparse at various search depths (solid lines) for Bur-0, Tsu-1 and Ler-1.

Our bubble detection algorithm was designed to detect more complicated bubble structures than the more conservative approach adopted by the Cortex bubble caller. To confirm this was the case, we used the same Bur-0, Tsu-1 and Ler-1 (50×) *Arabidopsis* reads as input to the latest version of Cortex_var (1.0.5.13) and verified the bubbles it output using the same method as for Bubbleparse. At optimal kmer size for each tool, Cortex_var called a lower percentage of the canonical SNPs than Bubbleparse for all values of the Bubbleparse depth parameter (Figure 4). For Bur-0 and Tsu-1, Cortex_var called around 40% of the canonical SNPs and Bubbleparse was able to locate over 80% at higher search depths. For Ler-1, Cortex_var called 20% of the canonical SNP list, while Bubbleparse called 38% at depth 0 and 53% at depth 2. As previously, this increased ability to find SNPs does bring increased false positives. For Bur-0 and Tsu-1, the false positive rate is comparable for Cortex_var and Bubbleparse, while for Ler-1, Cortex_var produced slightly less false positives (Figure S3).

Because Bubbleparse has been designed for reference-free situations, where a BWA/SamTools approach cannot be applied, comparison with these kinds of reference-based tools is problematic. In the previous section, we used BWA/SamTools derived SNP lists as a canonical set in order to assess Bubbleparse performance. In a separate experiment, we created a simulated mutant of the *E. coli* genome containing 100,000 randomly positioned SNPs. Using SimSeq, a tool created for the Assemblathon project [11], we produced 76 nt simulated Illumina reads of the reference *E. coli* and the mutant at approximately 20× coverage and using SimSeq's supplied Illumina GAIIx error profile. Both sets of reads were used as input to Bubbleparse and for comparison, we used BWA/SamTools to call SNPs based on the mutant reads aligned to the original *E. coli* reference. The BWA/SamTools combination was able to locate 86,275 of the SNPs, with only 1 false positive. Bubbleparse located 93,446 of the SNPs, with 12,917 additional false positives (Figure S4). This result underlines the ability of Bubbleparse to achieve comprehensive reference-free SNP recall, but also the importance of effectively ranking the SNPs to mitigate the effect of false positives.

### A ranking heuristic for the SNPs allows us to maximise the number of real SNPs detected

The number of bubbles collected in the graph search in all our tests was very large, indicating that the search is effective, but ultimately the numbers are far too large to be useful to an experimenter seeking to make a catalogue of useful SNPs. To allow for the accurate detection of graph structures that represent real SNPs and therefore to make the Bubbleparse results as useful as possible we assessed methods of ranking the bubbles. We collected attributes of the raw input sequence, namely: average coverage of the kmers in the bubble and quality scores given to the polymorphism by the basecalling software (total Q score is obtained by summing the quality values of the first divergent nucleotide of each path through the bubble), and attributes of the structure of the bubbles: the length of the path through the bubble (a bubble with all paths the same length and a path length equal to kmer size indicates a clearer SNP) and total difference (the amount that the mean path coverage percentage differs from an expected coverage %. For example, in a heterozygous vs homozygous experiment, we expect 100% coverage on one path, 0% on the other for the homozygous allele and 50%, 50% for the heterozygous).

**Figure 5:**
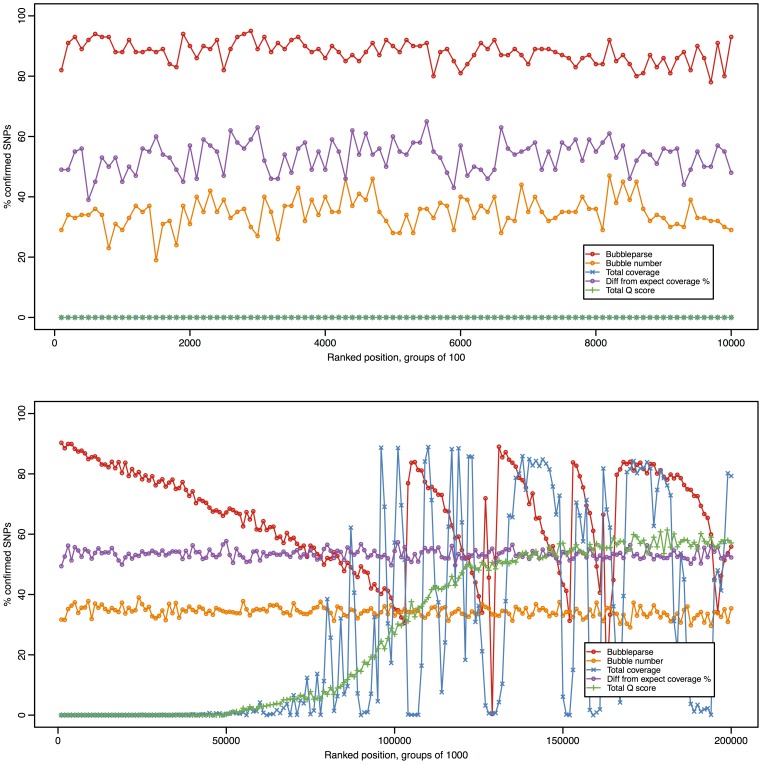
Efficacy of five different methods for ranking bubbles. In the top graph, moving down the ranked tables, groups of 100 bubbles were taken and compared with the canonical set to calculate the percentage of ‘true’ bubbles. In the bottom graph, groups of 1000 bubbles were taken, allowing the majority of the bubbles to be included. Ranking by the Bubbleparse heuristic produces a much higher true positive rate than any of the alternative methods over the top 50,000 SNPs. From around the 100,000 mark, the Bubbleparse line exhibits a saw shape, the peaks of which are caused by the individual constituents of the ranking heuristic. Note, in the top graph, the blue trace (total coverage) is obscured by the green trace, as both are almost 0.

We examined the bubble collections for the Ler-1 50× data at k = 21, search depth = 1, sorted the bubble table by each attribute in turn and calculated the proportion of real SNPs in sets of 100 bubbles, based on comparison with the 1001 genomes list. Arbitrary ranking of the table by the internal index of each bubble (bubble number) provided a baseline for detection of real SNPs throughout the bubble collection, the proportion of real SNPs in this ranking was constant through the bubble collection (Figure 5) indicating that the internal processing of bubbles prior to applying ranking does not output bubbles representing real or miscalled SNPs preferentially. The sequence based attributes were less useful than the arbitrary ranking at discriminating the real SNPs. When quality alone was used as a ranking metric the first 50,000 bubbles did not contain any real SNPs. From 50000 onwards, there was a steady increase in the accuracy up to a fairly consistent 60% thereafter (Figure 5). The poor initial accuracy may be an artefact of inflated total Q score due to higher than average kmer coverage such as that caused by sequence repeats. Ranking by coverage was also somewhat inefficient initially and as with the quality metric, there were no real SNPs in the first 50,000 bubbles; again this is likely caused by repeats. After the first 50,000 bubbles, there is erratic performance, with wildy varying highs and lows, which we believe are procedural artefacts due to repeats in the sampled genome and the SNP calling approach used (Figure 5B). The bubble structure based total difference score was much more effective than the sequence based measures and gave between 40–60% accuracy over the first 0–10,000 SNP calls, significantly higher than the baseline rate given by the match number. We reasoned that by specifying structural properties in our ranking before sequence properties we would preselect for more real SNPs and minimise the impact of repetitive regions, thus we developed a simple heuristic for ranking bubbles, the Bubbleparse ranking heuristic, which makes use of the following in order:

1. **The type classification of a bubble** – determined by the number of colours on each path through the bubble (Figure 1C). There is a separate ranked list for each bubble type.
2. **Contig length** – a bubble with contig length greater than or equal to the minimum contig length will be ranked higher than one with a contig length lower than the minimum. A minimum contig length is required for effective biological application - for example design of primers.
3. **Equality of path length** - a bubble with all paths the same length will rank higher than one with different path lengths, that is SNPs rank higher than Indels.
4. **Length of path through the bubble** - a bubble with all path lengths equal to kmer size is ranked more highly than one with path lengths not equal to kmer size.
5. **Mean coverage %** - bubbles where the mean coverage through each path is within a specified tolerance of the expected coverage % are ranked more highly than those with coverage outside the tolerance.
6. **Other incomplete paths** - a complete path through the bubble in a given colour is required for that colour to count towards the type classification; however, a partial colour path suggests uncertainty and causes the bubble to be ranked lower than one without any additional partial paths.
7. **Total Q score** - bubbles are ranked on total Q score, obtained by summing the quality values of the first nucleotide (ie. the first divergent nucleotide) of each path through the bubble. If no quality scores are available, this step is omitted.
8. **Combined coverage** – the combined coverage is obtained by summing the mean coverage values of each path through the bubble.

Accompanying pseudocode for this heuristic can be found in Supporting Methods S1. Based on comparison with the 1001 genomes SNP list, the Bubbleparse heuristic was successful at maintaining a very high accuracy of 80–90% SNP detection across the first 10,000 bubbles in our sample set (Figure 5). As expected, this accuracy rate declines with distance down the list, but a number of peaks can be observed, beginning just after the 100,000 ranked SNP. These are due to the individual components of the ranking heuristic and comprise predicted SNPs which, though having scored highly according to one measure, fail on another. For example, the first peak represents the most highly ranked of the SNPs which were dropped lower because they contained additional incomplete paths through the bubble; the peak illustrates that some of these were still good SNPs of the expected allele frequency, but the rapid drop-off indicates that it was still desirable for the algorithm to demote these bubbles. These discontinuities illustrate that there may still be scope for further optimisation of the ranking methodology in the future – perhaps through the use of machine learning techniques.

It was not possible to plot a ROC curve for the performance of the heuristic, as we do not make a binary distinction between bubbles due to SNPs and those due to other artefacts - which means that calculating true and false negatives has no meaning. Instead, the top of the ranked list represents bubbles for which there is high confidence that they are SNPs and are linked to the expected heterozygosity, while those at the bottom of the list represent bubbles with a low likelihood of being a real linked SNP.

To provide further confirmation of the tool's performance, we used Sanger sequencing to test the top 48 Bubbleparse ranked SNPs, as well as 3 sets of 16 SNPs that were placed at the 25%, 50% and 75% boundaries of all SNPs which were in contigs of 200 nt or greater (this length chosen for effective oligo design). We looked to see if the SNPs were in the 1001 genomes list by aligning contigs to the TAIR9 reference. Of the top 48, we were able to locate 39 SNPs in the 1001 genomes list; however, the sequencing successfully confirmed 46 of the SNPs, at the predicted position and with the predicted alleles (Table 1). The remaining 2 results were inconclusive, due to problems with the sequencing or the primers used. 45 of the remaining 48 SNPs placed at 25%, 50% and 75% in the ranked list were also confirmed, with 3 failed sequencing attempts (Table 1). Again there were SNPs confirmed by sequencing which we were unable to find in the curated list.

**Table 1:** Results of *Arabidopsis thaliana* Sanger sequencing.

Position in bubbleparse list	SNPs sequenced	Confirmed SNPs	Unconfirmed SNPs	Problems with sequencing	Number in curated list
Top	48	46	0	2	39
25%	16	15	0	1	13
50%	16	15	0	1	10
75%	16	15	0	1	11

From the ranked list of all SNPs predicted by Bubbleparse in contigs of over 200 nt, the top 48, as well as 16 from 25%, 50% and 75% down the list were tested with Sanger sequencing. This confirmed all but 5 as being real SNPs between Col-0 and Ler-1. The remaining five all had sequencing problems – such as the sequence ending before the SNP was reached – so are not confirmed as false postives.

We then examined the SNPs that were confirmed by sequencing but which we were not able to find in the curated list. Of these 23 SNPs, we were able to match 14 by relaxing the alignment criteria (Materials and Methods). This left 3 SNPs that we were unable to align to the reference and 6 of the 91 confirmed SNPs, which, though easily aligning to the reference, were not in the curated list. This result leads us to believe that Bubbleparse provides even higher accuracy than that indicated in Figure 5. Examining the locations of the 6 SNPs not found in the curated list, 4 turned out to be unknown proteins or non-coding genes (Table S2). There is insufficient data to draw firm conclusions, but these sorts of regions may contain repetitive elements that made detection of the SNPs harder.

### Bubbleparse is a fast and effective tool for detection of resistance-linked SNPs in unsequenced genomes

**Figure 6:**
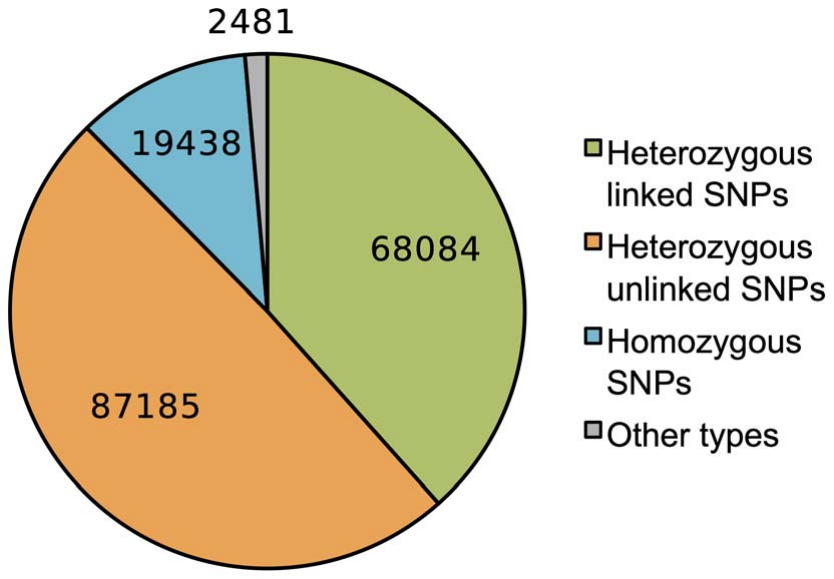
Types of *Solanum* SNPs discovered by Bubbleparse. Graph showing the types of SNPs discovered by Bubbleparse for the cross between *Phytophthora infestans* resistant *Solanum berthaultii* and susceptible *Solanum stenotomum*. Because of the nature of the cross, we expect to find heterozygous resistance-linked SNPs and Bubbleparse produced a list of 68,084 of these, from which we selected 27 for sequencing.

In proof-of-principle experiments, we tested the Bubbleparse method with bulked Illumina sequenced normalized cDNA from 30 resistant and 30 susceptible individuals of an F1 population from a cross between *Phytophthora infestans* resistant *Solanum berthaultii* and susceptible *Solanum stenotomum*. These plants were manually scored for resistance and found to have 50% resistance in the bulk resistant population, thus we expected the resistant genotype to be heterozygous and present at roughly 50%. We sequenced each normalized library at approximately 40× coverage (assuming 20,000 genes expressed with average size of 1500 bp and complete normalization). We ran Bubbleparse at a kmer size of 31 and with cleaning options set to remove tips of 100 nodes or less and any node of coverage of only 1. Bubbleparse identified 201,000 SNPs of different patterns of heterozygosity directly from reads (Figure 6). 68,000 linked SNPs (34 percent of all SNPs) at the expected heterozygosity pattern were found. The ranked result table was inspected and we selected 27 predicted SNPs, according to 4 different sets of criteria, for verification with Sanger sequencing. The first 10 of these SNPs were taken from the top of the Bubbleparse ranked list, representing SNPs with coverage ratios close to the expected heterozygosity and high absolute coverage; the remaining three groups were chosen from lower down the ranked list (within the top 22,000 out of the 68,000 linked SNPs at the expected heterozygosity) and were chosen because, while not presenting optimal results for all ranking parameters, they displayed either a close match to the expected ratio, high coverage, or high total quality score. Overall, 23 out of 27 (>85%) were confirmed as SNPs with 14 out of 27 as heterozygous SNPs directly linked to the resistant trait (Table 2), giving us a set of verified trait-linked SNPs in a straightforward analysis. Despite the small number of candidate SNPs we were resourced to analyse experimentally, validating more than 50% true positives across the different ranking priorities in this simple proof-of-principle experiment provides a strong indication that the method we present is effective. Interestingly, in our plant experiments we were unable to reliably identify short indels; theoretically indels should manifest as bubbles with branches of differing lengths, and although these could be found in the graph we were very rarely able to confirm them as real in subsequent sequencing experiments. This may indicate that the graph around indels is more difficult to search than that around SNPs.

**Table 2:** Results of *Solanum* Sanger sequencing.

	Type of SNP confirmed			
	Heterozygous linked SNP	Heterozygous unlinked SNP	Homozygous SNP	Total
**Maximise ratio and coverage**	^5^/_10_	^4^/_10_	^1^/_10_	^10^/_10_
**Maximise ratio**	^2^/_6_	^2^/_6_	-	^4^/_6_
**Maximise coverage**	^5^/_6_	^1^/_6_	-	^6^/_6_
**High total quality score**	^2^/_5_	^1^/_5_	-	^3^/_5_
**Total**	^14^/_27_	^8^/_27_	^1^/_27_	^23^/_27_

We chose 27 predicted SNPs to test with Sanger sequencing. These were chosen from the Bubbleparse ranked list according to four sets of criteria, with all SNPs placed within the top 22,000 of the 68,000 linked SNPs at the expected heterozygosity. The largest number, 10, were chosen from the top of the list, which contains bubbles which are close to the expected allele ratio and also of high coverage. A second group were chosen which were very close to the expected allele ratio, but not such good coverage. A third group had very good coverage, but were not so close to the expect allele ratio. Finally, a fourth group was chosen which had high quality scores, but not necessarily as close a ratio or as good a coverage as previous groups. Overall, we found a high rate of true SNPs – with 23 out of 27 sequences containing a SNP in the position predicted by Bubbleparse, of which 14 displayed the predicted heterozygous alleles. The group chosen from the top of the ranked list showed high accuracy, with 9 out of 10 SNPs confirmed, but high rates were also shown for the other groups, though sample sizes were small.

Our benchmarking and real data analyses show that Bubbleparse is a very fast and sensitive tool for creating catalogues of SNPs in a simple analysis in very short time frames. The experiment ran quickly so that in around 24 hrs, we were able to go from brand new Illumina sequence read data to a high quality set of heterozygous SNPs linked to a trait of agronomic importance that could be used as markers in downstream analyses. The Bubbleparse ranking scheme gives the tool extra discriminative power enabling us to find SNPs with very high accuracy, at levels that are easily comparable and competitive with alignment based methods for the examined classes of SNPs. The ranking is prioritised here for finding SNPs in the bubble collection of expected homo-/heterozygosity patterns but the Bubbleparse output is verbose and bubbles can easily be re-ranked for other purposes. This would make it possible to investigate alternative algorithms for ranking, perhaps based upon machine learning techniques, and in so doing, seek to remove the discontinuities found in the *Arabidopsis* ranking graphs reported in Figure 5. The flexibility of Bubbleparse makes it useful for a wide range of genetic backgrounds and sample types including complex crosses, multi-population samples and environmental metagenomes.

The Bubbleparse source code can be downloaded from https://github.com/richardmleggett/bubbleparse.

## Materials and Methods

### Validation of *Arabidopsis thaliana* predicted SNPs

The validation of the Bubbleparse predicted SNPs was performed using PCR amplification and Sanger sequencing. We designed oligos (Table S3) to match locations where SNPs were predicted.. 40 ng of total genomic DNA of *Arabidopsis thaliana* strains Col-0 and Ler-1 were used in a PCR reaction (94°C 30 seconds, 53°C 35 seconds, 72°C 60 seconds, 35 cycles) with non-proofreading DNA polymerase. 2 µl of the clean PCR reaction (∼20 ng) was used in the Sanger sequencing with either a forward or reverse sequence oligo. The chromatograms were analysed using the program DNASTAR Lasergene version 8.

To compare these SNPs with the curated list, we aligned the contigs to the TAIR9 reference genome using BLAST, looking for matches of 98% identity and 98% length. For the subsequent investigation into SNPs that were confirmed, but could not be found in the curated list, we relaxed the alignment criteria to 93% identity and 93% length in order to find the additional matches. Further relaxation of alignment criteria did not produce further matches.

### Potato plant material

We used 30 resistant and 30 susceptible plants from a cross between diploid parents, a resistant *Solanum berthaulltii* (PI1477731) [12] (Rauscher *et al.*, 2006) and a susceptible *Solanum stenotomum* (CGN19035).

### Potato sample preparation

Total RNA was extracted from two young leaves of each resistant and each susceptible plant by using TRIzol reagent (Invitrogen) from which 2.5 µg total RNA of each individual was bulked up to generate a bulked resistant (BR) and a bulked susceptible (BS) sample. PolyA+ RNA was extracted (Invitrogen) and double stranded cDNA (ds cDNA) was generated by using a SMART cDNA library kit (Clonetech). Next, we normalized the cDNA (duplex-specific nuclease, Evrogen) and after *Sfi*I (New England Biolabs) digestion, we selected fragments (>500 nt) with Chroma Spin 1000 columns. In order to prepare the samples for Illumina sequencing, we acoustically sheared the cDNA to fragments of average 200 bp (Covaris, settings: Duty cycle 20%, Intensity 5, cycles per burst 200, time 120 seconds) and ligated adapters to the fragments (Paired-End DNA Sample Prep Kit, Illumina). Subsequently, we size selected fragments (5% polyacrylamide gel) of 300 bp that were sequenced on the Illumina machine (76 nt read, paired-end). All methods were according the manufacturers protocols.

### Validation of predicted potato SNPs

To validate the outcome of the Bubbleparse SNP prediction, we designed oligos to match locations where a SNP was predicted (Table S4). 1 ng of ds cDNA, generated with the SMART cDNA library, was used in a PCR reaction (94°C for 30 seconds, 55°C for 30 seconds, 72°C 60 seconds, 35 cycles) with non-proofreading DNA polymerase and 1 µl of the reaction (5–10 ng) was used in Sanger sequencing with either a forward or reverse sequence oligo. The chromatograms were analyzed in VNTI ContigExpress (Figure S2).

## Supporting Information

Table S1 **Speed of execution.** The table provides typical execution times for assembly of de Bruijn graph, identification of potential SNPs and output of contigs for the Col-0 vs. Tsu-1 experiment. Times are for illustration only, as assemblies were carried out on a range of individual machines within a workload managed cluster. Increased numbers of kmers result in larger graphs, requiring more processing time to walk them. Increased levels of depth mean the bubble finding algorithm explores a much larger portion of the graph around a bubble, also resulting in increased execution time.
(DOC)

Table S2 **Sequenced SNPs not in the 1001 genomes list.** For SNPs that were confirmed by Sanger sequencing, but not in the 1001 genomes list, the table shows the position of alignment to the TAIR9 reference, as well as loci from the TAIR browser at www.arabidopsis.org.
(DOC)

Table S3 ***Arabidopsis thaliana*** **experiment oligos.**
(DOC)

Table S4 ***Solanum berthaultii*** **experiment oligos.**
(DOC)

Figure S1 **Kmer counts for different values of k in the Bur-0 and Tsu-1 datasets.**
(TIFF)

Figure S2 **Chromatograms for** ***Solanum berthaultii*** **experiment.** Typical chromatograms from Sanger sequencing confirmation of SNPs. In each case, the top graph represents the resistant bulk cDNA, the bottom graph the susceptible bulk cDNA and dotted lines indicate the predicted SNP location. (A) An example of a linked heterozygous SNP, with the resistant showing both a C and a G at the SNP position, while the susceptible shows only a G. (B) An example of an unlinked heterozygous SNP, where both resistant and susceptible show a C and a G at the SNP position. (C) An example homozygous SNP, with the resistant and susceptible each showing a single, different, nucleotide. (D) An example of an unconfirmed SNP, where there is no apparent nucleotide difference at the predicted SNP position.
(TIFF)

Figure S3 **SNP finding compared in Cortex_var and Bubbleparse.** Graphs showing percentage of canonical SNPs found and the percentage of predicted SNPs confirmed for Bubbleparse (BP, solid lines) at a range of search depths (d = 0,1,2,3) and Cortex_var (CV, dotted lines). (A) Bur-0 results for minimum BWA/SamTools pileup of 10 (left hand column) and 20 (right hand column). (B) Tsu-1 results for minimum pileup of 10 (left hand column) and 20 (right hand column). (C) For Ler-1, a curated SNP list was available, so only one column.
(TIFF)

Figure S4 **Bubbleparse SNP recall for synthetic** ***E. coli*** **.** Graph showing percentage of SNPs found by bubbleparse, percentage of true positive SNPs output by bubbleparse and percentage of false positive SNPs output for a simulated *E.coli* dataset. Input was a set of simulated reads from the *E. coli* genome and a second set of simulated reads from an *E. coli* genome containing 100,000 simulated SNPs. Both sets of reads were designed to mimic 76 nt Illumina reads of approximately 20× coverage. Bubbleparse was run with minimal cleaning (removing paths of coverage 1 or less and tips of 100 nodes or less) and search depth 1.
(TIFF)

Methods S1 **Implementation of bubbleparse heuristic.**
(DOC)

Methods S2 **Variation of coverage for Ler-1 experiments.**
(DOC)
