# Supplementary material for: Identifying and Classifying Trait Linked Polymorphisms in Non-Reference Species by Walking Coloured de Bruijn Graphs
Source: PLoS One. 2013 Mar 25;8(3):e60058. doi: 10.1371/journal.pone.0060058 (PMC3607606; doi:10.1371/journal.pone.0060058)
Supplement: Table S1 — Speed of execution. The table provides typical execution times for assembly of de Bruijn graph, identification of potential SNPs and output of contigs for the Col-0 vs. Tsu-1 experiment. Times are for illustration only, as assemblies were carried out on a range of individual machines within a workload managed cluster. Increased numbers of kmers result in larger graphs, requiring more processing time to walk them. Increased levels of depth mean the bubble finding algorithm explores a much larger portion of the graph around a bubble, also resulting in increased execution time. (DOC) [file pone.0060058.s001.doc]

| **Kmer size** | **No. of kmers** | **Depth 0 search time (hrs)** | **Depth 1 search time (hrs)** | **Depth 2 search time (hrs)** | **Depth 3 search time (hrs)** |
| --- | --- | --- | --- | --- | --- |
| 15 | 454,471,002 | 2.38 | 5.82 | 23.73 | 97.19 |
| 17 | 1,326,861,891 | 1.79 | 4.95 | 5.63 | 14.35 |
| 19 | 1,543,253,615 | 2.03 | 3.01 | 4.28 | 8.53 |
| 21 | 1,503,770,452 | 1.81 | 3.11 | 4.96 | 17.64 |
| 23 | 1,412,905,117 | 1.75 | 12.78 | 6.54 | 20.89 |
| 25 | 1,303,225,976 | 1.56 | 9.90 | 16.12 | 9.20 |
| 27 | 1,179,817,841 | 1.23 | 6.32 | 10.83 | 42.49 |
| 29 | 1,043,319,784 | 1.38 | 4.18 | 6.37 | 32.92 |
| 31 | 892,605,409 | 0.96 | 2.45 | 1.11 | 6.93 |
| Mean | N/A | 1.66 | 5.83 | 8.84 | 27.79 |
