# Supplementary material for: Identifying and Classifying Trait Linked Polymorphisms in Non-Reference Species by Walking Coloured de Bruijn Graphs
Source: PLoS One. 2013 Mar 25;8(3):e60058. doi: 10.1371/journal.pone.0060058 (PMC3607606; doi:10.1371/journal.pone.0060058)
Supplement: Table S2 — Sequenced SNPs not in the 1001 genomes list. For SNPs that were confirmed by Sanger sequencing, but not in the 1001 genomes list, the table shows the position of alignment to the TAIR9 reference, as well as loci from the TAIR browser at www.arabidopsis.org. (DOC) [file pone.0060058.s002.doc]

| **Bubble number** | **Position in SNP list** | **Chr** | **Position (TAIR9)** | **Locus** | **Description** |
| --- | --- | --- | --- | --- | --- |
| 23254 | 5 in top 48 | 3  3 | 6072012  6057785 | AT3G17740  AT3G17712 | Unknown protein  Unknown protein |
| 466413 | 8 in top 48 | 5  5 | 15675674  15689250 | AT5G39155  None given | TE gene  - |
| 80203 | 20 in top 48 | 5 | 25562702 | AT5G63870 | ATPP7, PP7, SERINE/THREONINE PHOSPHATASE 7 |
| 338278 | 6 in 50% | 1  1 | 23298102  23418441 | AT1G62900  AT1G63140 | S-adenosyl-L-methionine-dependent methyltransferase  O-methyltransferase family protein |
| 304692 | 8 in 50% | 4 | 17193550 | None given | is known SNP in col-0, Tsu-1 |
| 237963 | 3 in 75% | 3 | 4645660 | AT3G14025 | pseudogene of scarecrow transcription factor family protein |
