# Supplementary material for: Identifying and Classifying Trait Linked Polymorphisms in Non-Reference Species by Walking Coloured de Bruijn Graphs
Source: PLoS One. 2013 Mar 25;8(3):e60058. doi: 10.1371/journal.pone.0060058 (PMC3607606; doi:10.1371/journal.pone.0060058)
Supplement: Table S3 — Arabidopsis thaliana experiment oligos. (DOC) [file pone.0060058.s003.doc]

Top 48

| **Bubble No.** | **Forward oligo 5' - 3'** | **Reverse oligo 5' - 3'** |
| --- | --- | --- |
| 307512 | AACAGAGATGGAAATAGTCGA | TCGAGAGCGTTTTCTTCCTC |
| 3688 | ACCGCGACAATCCTAACCTT | CCATCTCCAGGTCGTGAGTT |
| 57243 | GTGCATTGTCAGAGGAATCTA | CGATTCCAAGGGAGACTCAA |
| 262168 | GCCATCTCCTCTGCTTCTTC | CTTCAGCATTTCTAAGAGCC |
| 23254 | GCCAGCCATGCCTTTGCATC | CCATGGAACGCAACAAGAAT |
| 366521 | GTCTGGGATTTTGGAGTCA | AGGGGGTCGAATCAGAGACT |
| 206254 | CTCCGCCATCTCATCTTCTC | AGGACAACGCAGTTGAGGTTTG |
| 466413 | GACAGCGCATCCAAAGACTC | GGATTCGTCTAGACCCATG |
| 60726 | GAGGATGAGAGCGCTGAGA | TTGTGTCGATTGACATTCGAG |
| 367584 | TGTAACCCGCTTTCTCCATC | GTTATCACTAATATGCCGATTG |
| 110574 | GCTCGTGCTATGTCTTCC | GACGATGAAAGGACTTCCA |
| 77647 | CTCAGGTCAATCGTCACAA | CGGATGTGTTTTTCGCCTAT |
| 278479 | CTGACGGTGGACCCACCG | TGATTTCATCGGCGGTAAGC |
| 364553 | CTTGTCGCAGTAGCCAAGAA | TCGCACCTCCATAGATTTT |
| 146309 | TCTGCTTGGCTCTTCCATAA | TTCCCTGCAGTTATCGGTTC |
| 100177 | ACTGACAAGGAAGCTGCTC | ACGGCTGAGCCTTTGAGTT |
| 28107 | AGATTCAATAATGGAACCAGT | TTCGTTGAGTTTGGTTACAGGA |
| 78572 | CGAACGGATCTTCTCCAAG | CTAACAGTTGAAGCTCAAGCT |
| 240784 | TGTTTCTGTTTGCTCTTGGTTT | CTGCAAAGCAAGTGGATCGC |
| 80203 | GTTCTGGTGGCTTTGATCG | AACACCGGTTTTGACAAGGA |
| 457620 | GTTTGTAACGCGATTCGTGA | CCAATAGCTTGTAGTTGCTTAGA |
| 35302 | AACCAAGCTTGAGACGAT | CAGATTCCAAGCTCAAGCATG |
| 452652 | CCTCGAGTTGTGTCAGAGCA | GAGATTTGGCATAACTAAGTG |
| 26770 | CTTGCATTTGTTCCAGTCCA | GGACAAGCTCTCTCTCCTTATCT |
| 304338 | GTGGGGAACAGGAGAATTGA | CAAGGGAATGTGTTGGAGTG |
| 342867 | GAGGCCTTTCGGTGTGTG | CGACCAAGCTCTGAATTGA |
| 8345 | AATGGAATGATGCTGAGAAG | TGGATTGGTCAATCAACGAG |
| 116841 | TCATCGCTCTGATCGCTAAA | ATCCGTTTCGTCTTCTTCTCTG |
| 393133 | CTCCAAGGCTGGAAGGAC | CACAGCGGAATAAGTCCAA |
| 391407 | ATCTCTCACCTTGGGAGAGA | GCGCCAGGGCTAATGCTTGT |
| 180479 | TGCCAACATTTCCCTTGATT | TGTTTGTTGTGGATTTGAGAT |
| 162866 | AGTTTTGGGGAACAGGCTCT | CCAGGTAAACACCAATGTATAC |
| 378157 | CATAGACCGAGGAGAGACC | CCTCTACGTAGTGAATTTTCCC |
| 211795 | ACAGCCTTTCACCTTCATGG | TTTGATGAGCAAAAGAGACGAA |
| 42311 | CTTGCAATGCAAGCAAGAG | TGAAGTTCTGATTTCCTTGATT |
| 322385 | GCAGTGCAATTGAGTGATAA | GCTGTGAGTTCAGAGTCTTCT |
| 157217 | GACTCTAAAACATCACCAGCG | TGATTCCGGTGTAAACGTCA |
| 348591 | GCTCTTCCTCTACTCACCAAT | CTGTTATCCTCCTCCCTTTC |
| 441390 | TGCTCTTCCTCTACTCACCA | CTGTTATCCTCCTCCCTTTC |
| 366101 | CTTGGTTAAATAGGTTATAGCCTG | CATGGTGAAAGTGACTGTTATACTT |
| 277390 | AAGGTGTGATGGATAACCG | TCTCGAGGTAGCTCTTGGTC |
| 210936 | GATCCGACGATGTTTGATC | CGTGGCCGAGCTTCCCCG |
| 405008 | CCTAATTAACCCCAAAACATT | CCTGCAGAGAGGGACGCGAC |
| 275558 | GGCAACTTTACCATGAATCAC | CTTGATGTTGAAATCGACA |
| 61856 | CAGGAATCGAAGTGAAAACC | ATGCGTGGTGATCGTGAAA |
| 212485 | GCTCGTGCCTGTCCAAAGG | GTGTTCATGTGTACAGAAGGC |
| 209613 | TAACTCTAGACATCCTGAGCAAC | CCCAAGACCCCACTGTTTT |
| 119103 | ACTCTTGTCACCACAACACC | CCTCGGTTTTAATCGCGGAG |

25% down

| **Bubble No.** | **Forward oligo 5' - 3'** | **Reverse oligo 5' - 3'** |
| --- | --- | --- |
| 221742 | TGGATATTTGCAATTTTTAGAGTCA | CCATGGTCCAACTCCATTTC |
| 242193 | TCTGGAGCAAGTTTCGAGTTC | TGGTCACATTCGAAACAAGG |
| 268954 | AGGGGGTGGTCAGCTCTAAT | AAAAATTCAGACGCACAAAAA |
| 83113 | CCCGAACACGAACATAGTCC | TTTAGAAGAAATTATGAAAATTGGA |
| 89959 | AGGATGCCTACAGCTGGGTA | CCCTGCAAAGCAAAGAATGA |
| 106507 | TTGGATACAAGTTGGCTGGTT | TGTTCACAGAAGATCCATTCTCA |
| 327186 | TTGATGCACAAAAAGCAAAA | TGCTTGCGTATGTACCCAGAT |
| 389442 | TCATCAGTGAACAATTCGATACG | AGCAATCAGAATCCTTCCAA |
| 499551 | CCATAAAGCATATTTACATAATCAGAAGA | CCAAAACCAGATGCTGAACA |
| 232131 | TTATAAAAATTCTAATGCTCACAGT | GGAGAATCGTATTGTCCTGTCA |
| 253284 | AGCTCCATTGCTACCCATACA | GCAGCCACAAGATCTACTTTCA |
| 276480 | AGGCACATATGGGTCATGAAG | GACTCATGAGCTATGAACAAAACAA |
| 424302 | AGTCGAATTATGAAATAAAACCAT | AAGTGTGCAACGCAATCAAC |
| 425616 | GAATCCACTAAAATCTATCCACGA | AAGTGTGCAACGCAATCAAC |
| 333622 | GACTTTGTTATCCTCCAAGCTG | CGCATTCTAAGTTAAGTTAAACAAA |
| 345292 | AAAGAACGTGCATGCATTAGG | TTTTGAGATGCTAAAATGCTTG |

50% down

| **Bubble No.** | **Forward oligo 5' - 3'** | **Reverse oligo 5' - 3'** |
| --- | --- | --- |
| 128356 | GAAGCGGACCTTGAAGTGAG | AAAAGGGGTAGTTATGCAATTT |
| 190889 | CATGAACATATAATCGTGAAATGC | TGAGGATTAACATGCGGTGA |
| 218175 | TTTCTATTTATGTGAACGATGATG | TTGCAGCTCATGATTTGTAAAAG |
| 306921 | TGCCGATTGACTATTGGGTTA | CACCATCTCCGGATCTTCTC |
| 308551 | TCAATGGCAAGTTAAGTGAGAAA | TTCATTGCTCGTTTCCATAGG |
| 338278 | GATGGCATCTCCTTTTGGAA | TTCCAAGTATCCTCATATTAAAGG |
| 2304 | GGTGAAGAATTTATTTTATTGACATTT | ATATGAATCCGCGTGGAGAA |
| 304692 | GCGCTTGATGATATTTAATTCG | TTCATTTTTGGTTTATCCATTT |
| 306166 | CCGCTTCTAAGAAAATTCACATT | TGAGTTTTGATAACCCTTGAGTTT |
| 174444 | TTCAAACTCGAATCCTTTCGTT | CAGAACTTAGTATTGGTTGAGTCACA |
| 234636 | TGTATTGGGCATTTACTTGGTT | CCATACTTCACCCATTTTCACC |
| 236178 | AGCCTGTCGCGGTTTCAG | CGATCCTCTCCAACAGAAACAT |
| 317222 | GCTGAGTATGATAATAATTGCATAAGAA | TTTTCTGATGAGTCCAGTGACA |
| 459675 | GAGGAAATCAGTGCAATCCA | TGCATCAAATAAACTTTAGAAGG |
| 79594 | CACAATCAAAAACTGATAACGACA | GGGTAACATTGCCGTAAATGAA |
| 149368 | TTTAGGTCAGCGATATGGTCA | GAAAATGACAATCATGTGATGC |

75% down

| **Bubble No.** | **Forward oligo 5' - 3'** | **Reverse oligo 5' - 3'** |
| --- | --- | --- |
| 221801 | TCAAGAGGAGATTGCTTGGTT | CGTAATCTTGCCTCCTCAGA |
| 390110 | TCTGTTCTGACTTGTGGTGGTT | AAATTAAACGACGGGGAAAAA |
| 237963 | GGTTAGATTGTGATTATGAATGTTGC | GATGGAGATACCTTTGCTGGA |
| 125223 | GGAGCACCTTTGCTCACTCT | GCTGCTATAGTCGGGATGCT |
| 326897 | GGACACATTCCCTCCACATC | AACACCTTCTTCCCCTTCAAA |
| 491828 | TGAGTACAAATTCCGAGGATG | AGATACGCCGTCGTTAATCC |
| 42066 | TCTTCTATATGCTCATTCAAAATATC | GAGGAACAACTTAAGTGCCTACG |
| 216319 | GAAACTTTTCGGTGGATAGTTAAA | TTTGTGTTTTGGTTGGTGGA |
| 142396 | TGGCTTCCACTGATAAACCT | CTCGATTTGGACTATACTTGTGC |
| 263146 | GGTCGATTGATGGAAGATGG | TGGTAAGTGTTGTAGCAAATCCA |
| 161389 | CATTATCCTCAAGATGAGAAACA | TTGATTTGTCTTGTCCTGAAA |
| 237671 | AGAAATTCACTGCCCCAAAG | GTAATTTCGGAAAGTTCAAATA |
| 420769 | TGCGTTGGATCTAGTTTGGA | AGGTGGAGCTGATCACCAAG |
| 203894 | TCTGTTCACAAAATTTCTGTATATT | TGCAGATCTAATTGTAGGGAGGA |
| 351452 | CTGGAATAGCAGAAGACTTTTCA | CATCAGGTTCCCTATTCAACTG |
| 400108 | GCTACACAATACTAAGTCCATCTTTTG | GTCGGGTAATGGTCAAGGAA |
