# Supplementary material for: Identifying and Classifying Trait Linked Polymorphisms in Non-Reference Species by Walking Coloured de Bruijn Graphs
Source: PLoS One. 2013 Mar 25;8(3):e60058. doi: 10.1371/journal.pone.0060058 (PMC3607606; doi:10.1371/journal.pone.0060058)
Supplement: Table S4 — Solanum berthaultii experiment oligos. (DOC) [file pone.0060058.s004.doc]

| **Bubble No.** | **Forward oligo 5' - 3'** | **Reverse oligo 5' - 3'** |
| --- | --- | --- |
| 113252 | ttggggtgccattattgccac | CCACTGCTCCATTAATAAAATC |
| 132215 | cggcagtgatagcaactcctc | GGATGAGCAACTCCATTTCAGG |
| 132363 | acccctagttcaaactcttctg | GTCTTGACAGCCTCAAGTGAAAG |
| 13229 | gtaaagtaaatggtacctttg | TCTTCAAAGCCCAGCAGAATT |
| 141233 | agtaagctgcagttgaagtcc | GTCAGGCTTAATGAGGAGTGG |
| 148524 | cacttgagcgtctacacagc | CCAAAACCACACAAAAGTCCTG |
| 151027 | tcagtctgataaaacattgac | TAGCCCTGCATATTCCTGTC |
| 15325 | ctatagctttattgggagtg | GGAAGACCGACAACTTTTGAAC |
| 153787 | cgccttcagctaccttgtagc | AAGAACTGTTATTCCAGCTG |
| 155023 | gtggcaaatgtagcggaatcag | CTCTGATGAGATTTCCAAGGCC |
| 165799 | agaaacaagcttctttagccc | GGCGGTTTTAATGCGCAGAGTC |
| 168512 | gaataagggggctaagattgg | ATCCCGCACTACTACGC |
| 169101 | gttggagcttgttgatttgcc | CTCACCTGAAGTGCATGGACAG |
| 18052 | gagtggcgactctgatcagctc | AGATCATGATGTGTTAGGAATGGAG |
| 188648 | aaagcttatcagttagaaacc | TTGTTTTCACTAGTAAAACATCTTC |
| 192706 | cttggtggtgtaacctcaaaag | ATACATACTACTACCAACAACG |
| 198471 | ttggtgctttctttggaagggg | GAGAGTTAAATTTGTCATGGGTC |
| 29953 | ggactgaaaagttacaatatg | GAAACACTTCAATTCACTTTAGC |
| 41842 | ggaagatatcctcacgttatc | TAAGCCTTGAGATATCAGAGC |
| 50665 | gttgtggattttgatattgac | AGCTGGCCTTTGCAACAGGG |
| 58180 | tggaagagtattgattcaatc | GGAACATTTTCCTCAATGTAT |
| 65541 | ctggctcatgcagctgaatcaa | AAAGAAAGTTGTTTCTCTAAAAAC |
| 66875 | tttcccatctctctagcaatg | TGTAAGCAGAATTTGAAAGCC |
| 73069 | gtagtcatagcatgtcataatg | GAGGTAATTCCTGAAATAAGGG |
| 75486 | gacattcctggttgctgagg | TAGTAGCCTTAGTGAAATACATG |
| 79064 | ggcttgacctaaaattgaatttg | GAATTCGTCGGGGACATGGTC |
| 88677 | tcatactgtcggggtgatga | ATTACGTACAACAAAATCTCC |
| 92211 | aatgcttgggaagtctaagaac | CCTTTTCCAACTAACATACATC |
| 93799 | ccagtcgcaaatctgttgtac | TTTGATCTCCGGGAATTTATG |
| 96700 | gtcatctggatgcaaagaatgc | GCGATGGCCCACACAACCTC |
