# Supplementary figures and images for: Identifying and Classifying Trait Linked Polymorphisms in Non-Reference Species by Walking Coloured de Bruijn Graphs
Source: PLoS One. 2013 Mar 25;8(3):e60058. doi: 10.1371/journal.pone.0060058 (PMC3607606; doi:10.1371/journal.pone.0060058)

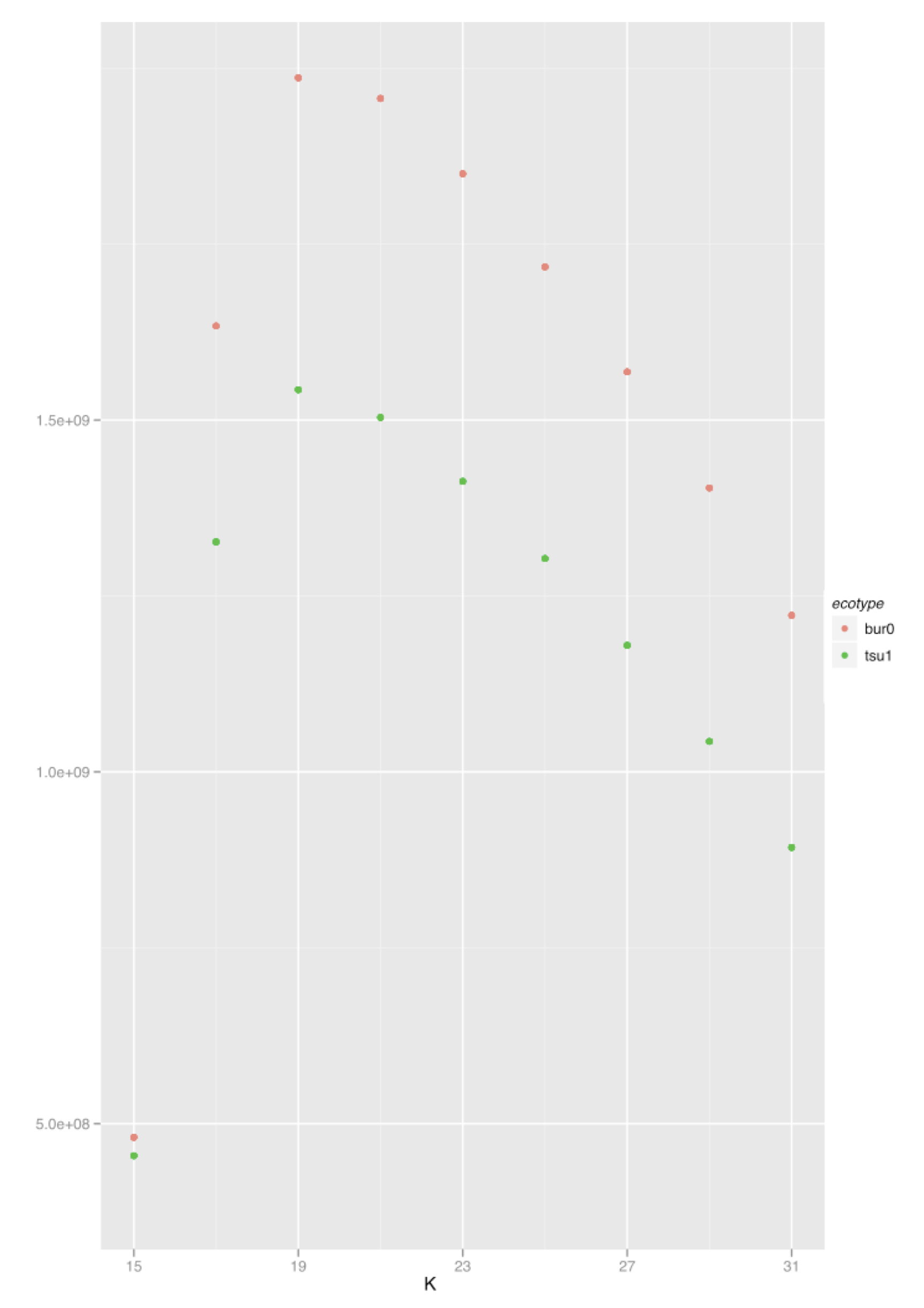

Supplement: Figure S1 — Kmer counts for different values of k in the Bur-0 and Tsu-1 datasets. (TIFF) [file pone.0060058.s005.tif]

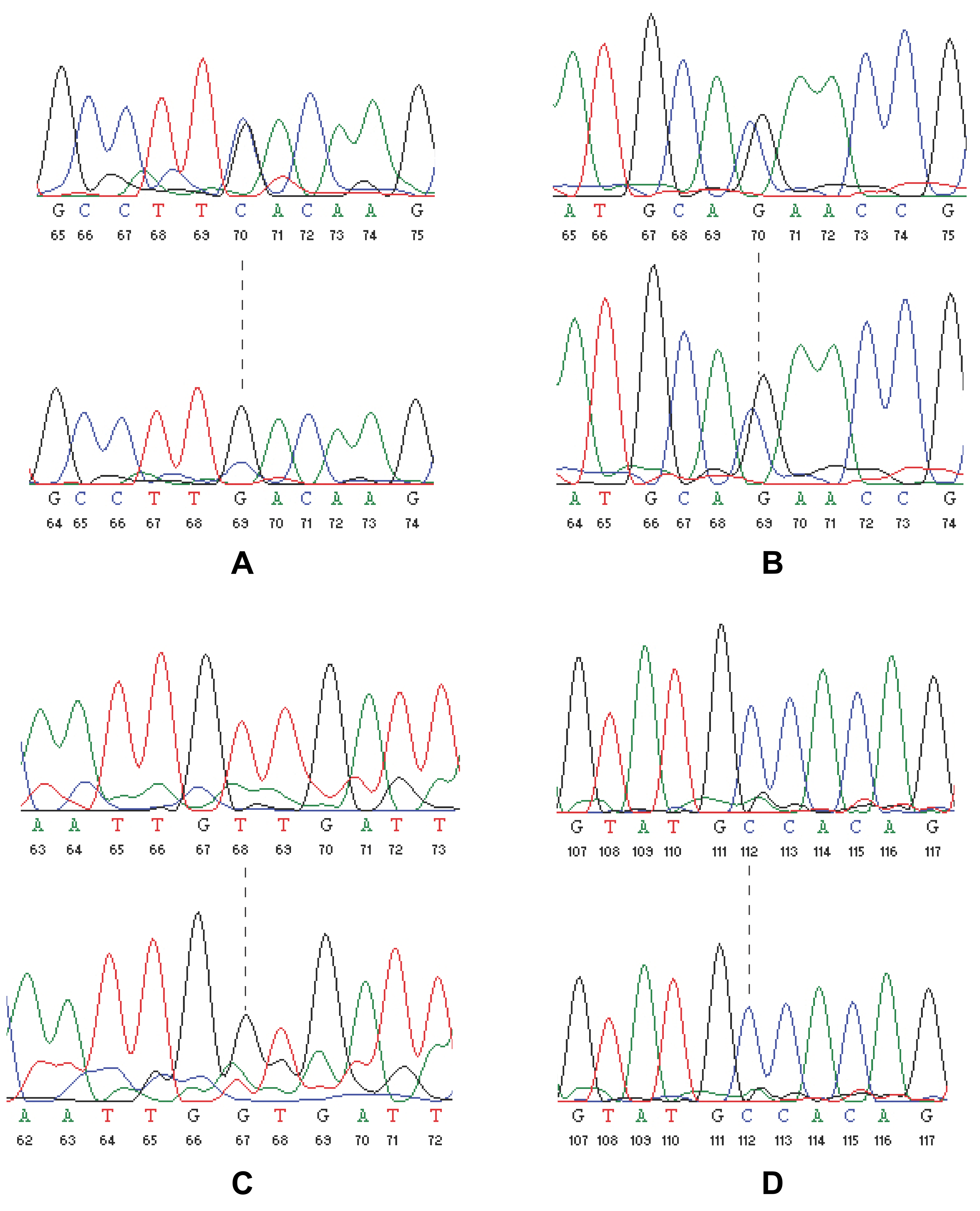

Supplement: Figure S2 — Chromatograms for Solanum berthaultii experiment. Typical chromatograms from Sanger sequencing confirmation of SNPs. In each case, the top graph represents the resistant bulk cDNA, the bottom graph the susceptible bulk cDNA and dotted lines indicate the predicted SNP location. (A) An example of a linked heterozygous SNP, with the resistant showing both a C and a G at the SNP position, while the susceptible shows only a G. (B) An example of an unlinked heterozygous SNP, where both resistant and susceptible show a C and a G at the SNP position. (C) An example homozygous SNP, with the resistant and susceptible each showing a single, different, nucleotide. (D) An example of an unconfirmed SNP, where there is no apparent nucleotide difference at the predicted SNP position. (TIFF) [file pone.0060058.s006.tiff]

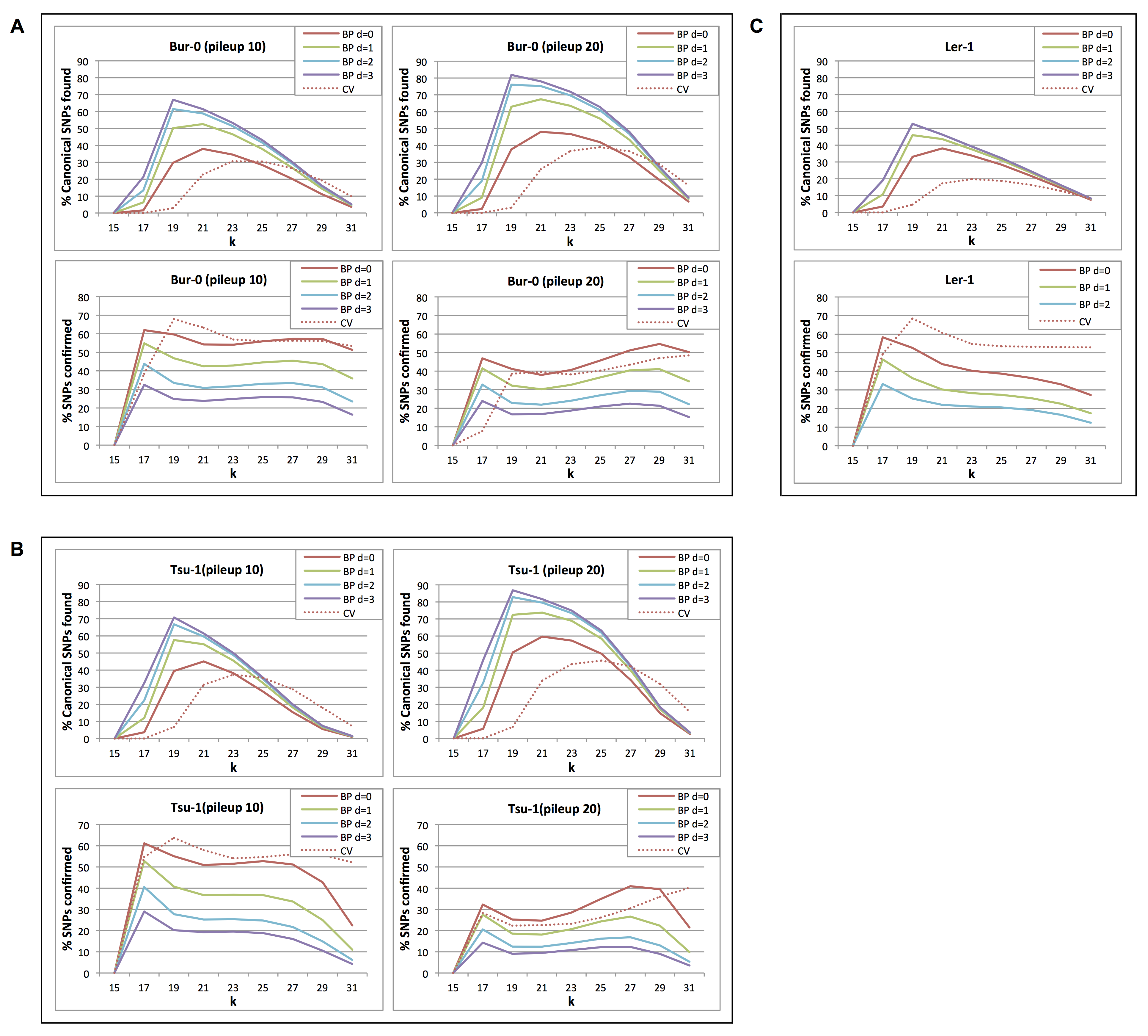

Supplement: Figure S3 — SNP finding compared in Cortex_var and Bubbleparse. Graphs showing percentage of canonical SNPs found and the percentage of predicted SNPs confirmed for Bubbleparse (BP, solid lines) at a range of search depths (d = 0,1,2,3) and Cortex_var (CV, dotted lines). (A) Bur-0 results for minimum BWA/SamTools pileup of 10 (left hand column) and 20 (right hand column). (B) Tsu-1 results for minimum pileup of 10 (left hand column) and 20 (right hand column). (C) For Ler-1, a curated SNP list was available, so only one column. (TIFF) [file pone.0060058.s007.tiff]

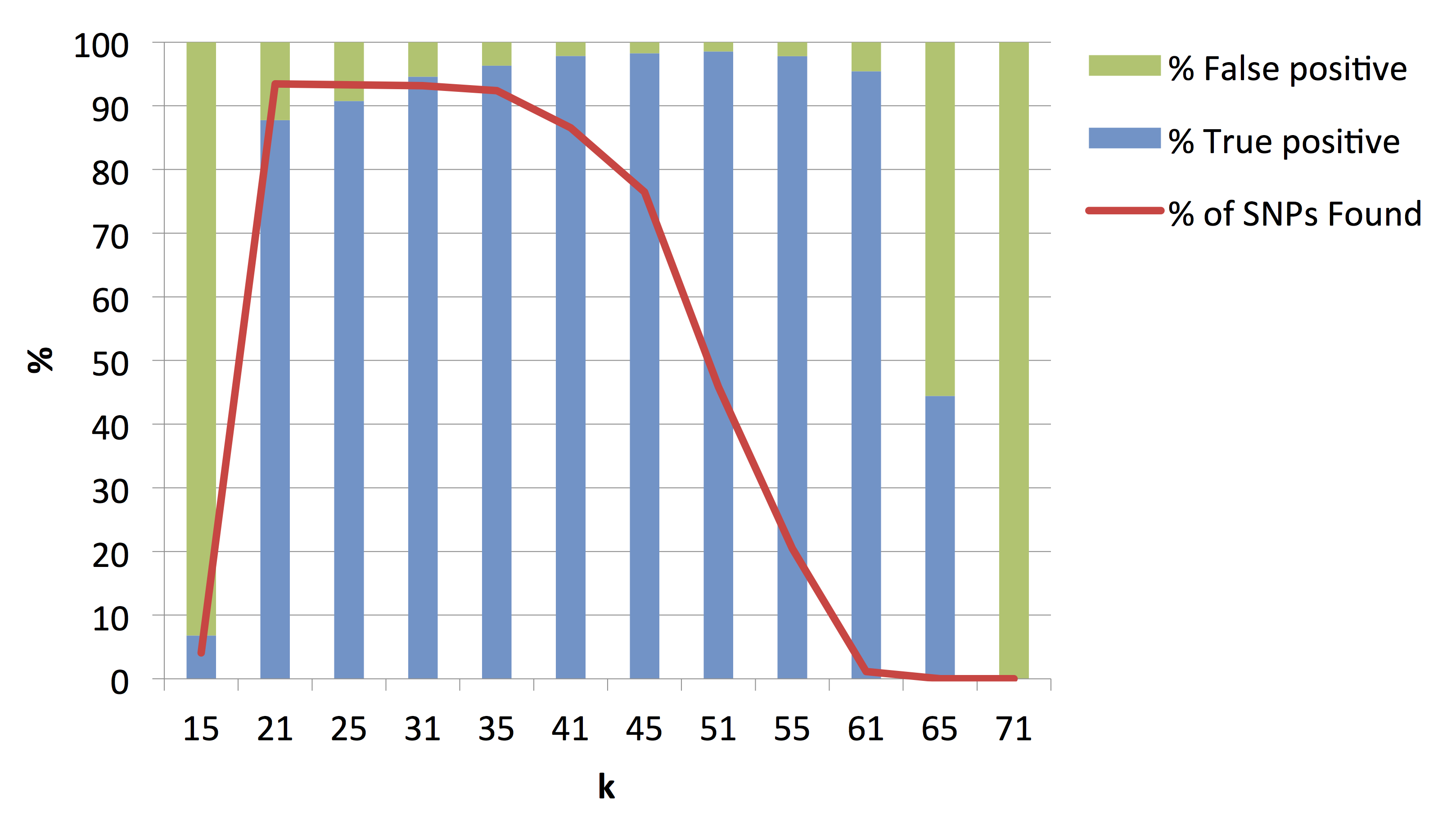

Supplement: Figure S4 — Bubbleparse SNP recall for synthetic E. coli . Graph showing percentage of SNPs found by bubbleparse, percentage of true positive SNPs output by bubbleparse and percentage of false positive SNPs output for a simulated E.coli dataset. Input was a set of simulated reads from the E. coli genome and a second set of simulated reads from an E. coli genome containing 100,000 simulated SNPs. Both sets of reads were designed to mimic 76 nt Illumina reads of approximately 20× coverage. Bubbleparse was run with minimal cleaning (removing paths of coverage 1 or less and tips of 100 nodes or less) and search depth 1. (TIFF) [file pone.0060058.s008.tiff]
