# Supplementary material for: Identifying and Classifying Trait Linked Polymorphisms in Non-Reference Species by Walking Coloured de Bruijn Graphs
Source: PLoS One. 2013 Mar 25;8(3):e60058. doi: 10.1371/journal.pone.0060058 (PMC3607606; doi:10.1371/journal.pone.0060058)
Supplement: Methods S1 — Implementation of bubbleparse heuristic. (DOC) [file pone.0060058.s009.doc]

# Methods S1: Implementation of bubbleparse heuristic

Bubbleparse analyses each set of bubble contigs, calculates various statistics and classifies the type of each bubble. For each bubble type (e.g. ‘2,1’ or ‘1,1’), it uses the C language qsort function to sort the list of bubbles according to our heuristic. The sort works by comparing adjacent pairs of bubbles and calling on a comparator function to decide if one is ranked higher than the other, or if both are the same. The following provides pseudocode for this function which would allow the heuristic to be independently implemented.

**Input:** A and B are two bubbles containing 2+ paths

**Output:** either A_ranked_higher, B_ranked_higher or A_and_B_same

if (contig_length(A) < minimum_contig_size && contig_length(B) >= minimum_contig_size) then

return B_ranked_higher

else if (contig_length(B) < minimum_contig_size && contig_length(A) >= minimum_contig_size) then

return A_ranked_higher

endif

if (path_lengths_are_same_length(B) and not path_lengths_are_same_length(A)) then

return B_ranked_higher

else if (path_lengths_are_same_length(A) and not path_lengths_are_same_length(B)) then

return A_ranked_higher

endif

if (path_lengths_are_same_length(A) and path_lengths_are_same_length(B)) then

if (bubble_path_length(A) != kmer_size and bubble_path_length(B) = kmer_size) then

return B_ranked_higher

else if (bubble_path_length(B) != kmer_size and bubble_path_length(A) = kmer_size) then

return A_ranked_higher

endif

endif

if (coverage_ratio_within_tolerance(A) and not coverage_ratio_within_tolerance(B))

return A_ranked_higher

else if (coverage_ratio_within_tolerance(B) and not coverage_ratio_within_tolerance(A))

return B_ranked_higher

endif

if (has_additional_broken_paths(A) and not has_additional_broken_paths(B))

return B_ranked_higher

else if (has_additional_broken_paths(B) and not has_additional_broken_paths(A))

return A_ranked_higher

endif

if (total_quality(A) > total_quality(B))

return A_ranked_higher

else if (total_quality(B) > total_quality(A))

return B_ranked_higher

endif

if (combined_coverage(A) > combined_coverage(B))

return A_ranked_higher

else if (combined_coverage(B) > combined_coverage(A))

return B_ranked_higher

endif

return A_and_B_same
