# Supplementary material for: Identifying and Classifying Trait Linked Polymorphisms in Non-Reference Species by Walking Coloured de Bruijn Graphs
Source: PLoS One. 2013 Mar 25;8(3):e60058. doi: 10.1371/journal.pone.0060058 (PMC3607606; doi:10.1371/journal.pone.0060058)
Supplement: Methods S2 — Variation of coverage for Ler-1 experiments. (DOC) [file pone.0060058.s010.doc]

**Methods S2**: Variation of coverage for Ler-1 experiments

Coverage was varied by removing FASTA input files from the assembly process, as indicated in the following table. All reads were downloaded from http://1001genomes.org/data/MPI/MPISchneeberger2011/releases/current/Ler-1/Reads/. As Cortex does not currently make use of pair information, no advantage is obtained by including both pairs of paired end or mate pair libraries.

|  |  | **Included in** | | | | |
| --- | --- | --- | --- | --- | --- | --- |
| **Read file** | **Bases** | **340x** | **170x** | **100x** | **50x** | **40x** |
| Paired-end-Lib2/s_88_8_single_sequence.fq | 4779345 | Y | Y |  |  |  |
| Paired-end-Lib2/s_88_8_2_sequence.fq | 292105609 | Y | Y |  |  |  |
| Paired-end-Lib2/s_88_8_1_sequence.fq | 337374697 | Y |  |  |  |  |
| Paired-end-Lib2/s_88_6_single_sequence.fq | 5568561 | Y | Y | Y | Y |  |
| Paired-end-Lib2/s_88_6_2_sequence.fq | 384951476 | Y | Y | Y | Y |  |
| Paired-end-Lib2/s_88_6_1_sequence.fq | 443711000 | Y |  |  |  |  |
| Paired-end-Lib2/s_88_5_single_sequence.fq | 5816300 | Y | Y |  |  |  |
| Paired-end-Lib2/s_88_5_2_sequence.fq | 336559464 | Y | Y |  |  |  |
| Paired-end-Lib2/s_88_5_1_sequence.fq | 407411125 | Y |  |  |  |  |
| Paired-end-Lib2/s_21_8_single_sequence.fq | 5970586 | Y | Y | Y |  |  |
| Paired-end-Lib2/s_21_8_2_sequence.fq | 1377694094 | Y | Y | Y |  | Y |
| Paired-end-Lib2/s_21_8_1_sequence.fq | 1371341408 | Y |  |  |  |  |
| Paired-end-Lib2/s_21_3_single_sequence.fq | 13957811 | Y | Y |  |  |  |
| Paired-end-Lib2/s_21_3_2_sequence.fq | 1290787844 | Y | Y |  |  |  |
| Paired-end-Lib2/s_21_3_1_sequence.fq | 1384435780 | Y |  |  |  |  |
| Paired-end-Lib2/s_21_2_single_sequence.fq | 9970768 | Y | Y | Y | Y |  |
| Paired-end-Lib2/s_21_2_2_sequence.fq | 1216918572 | Y | Y | Y | Y | Y |
| Paired-end-Lib2/s_21_2_1_sequence.fq | 1197205100 | Y |  |  |  |  |
| Paired-end-Lib2/s_20_6_single_sequence.fq | 6956550 | Y | Y |  |  |  |
| Paired-end-Lib2/s_20_6_2_sequence.fq | 378034666 | Y | Y |  |  |  |
| Paired-end-Lib2/s_20_6_1_sequence.fq | 384974412 | Y |  |  |  |  |
| Paired-end-Lib1/s_88_4_single_sequence.fq | 23559285 | Y | Y | Y |  |  |
| Paired-end-Lib1/s_88_4_2_sequence.fq | 1240807338 | Y | Y | Y |  |  |
| Paired-end-Lib1/s_88_4_1_sequence.fq | 1202360071 | Y |  |  |  |  |
| Paired-end-Lib1/s_88_3_single_sequence.fq | 31196308 | Y | Y |  |  |  |
| Paired-end-Lib1/s_88_3_2_sequence.fq | 1416071283 | Y | Y |  |  |  |
| Paired-end-Lib1/s_88_3_1_sequence.fq | 1374827470 | Y |  |  |  |  |
| Paired-end-Lib1/s_88_2_single_sequence.fq | 34313885 | Y | Y | Y |  |  |
| Paired-end-Lib1/s_88_2_2_sequence.fq | 1427090077 | Y | Y | Y |  |  |
| Paired-end-Lib1/s_88_2_1_sequence.fq | 1388458556 | Y |  |  |  |  |
| Paired-end-Lib1/s_88_1_single_sequence.fq | 32466646 | Y | Y |  |  |  |
| Paired-end-Lib1/s_88_1_2_sequence.fq | 1323119512 | Y | Y |  |  |  |
| Paired-end-Lib1/s_88_1_1_sequence.fq | 1295527418 | Y |  |  |  |  |
| Paired-end-Lib1/s_21_4_single_sequence.fq | 11185794 | Y | Y | Y |  |  |
| Paired-end-Lib1/s_21_4_2_sequence.fq | 1384878250 | Y | Y | Y |  |  |
| Paired-end-Lib1/s_21_4_1_sequence.fq | 1660684812 | Y |  |  |  |  |
| Paired-end-Lib1/s_21_1_single_sequence.fq | 11472035 | Y | Y |  |  |  |
| Paired-end-Lib1/s_21_1_2_sequence.fq | 1633803522 | Y | Y |  |  |  |
| Paired-end-Lib1/s_21_1_1_sequence.fq | 1646126061 | Y |  |  |  |  |
| Paired-end-Lib1/s_20_4_single_sequence.fq | 38075614 | Y | Y | Y | Y |  |
| Paired-end-Lib1/s_20_4_2_sequence.fq | 1303293973 | Y | Y | Y | Y | Y |
| Paired-end-Lib1/s_20_4_1_sequence.fq | 1271321584 | Y |  |  |  |  |
| Paired-end-Lib1/s_20_3_single_sequence.fq | 115605899 | Y | Y |  |  |  |
| Paired-end-Lib1/s_20_3_2_sequence.fq | 1244361994 | Y | Y |  |  |  |
| Paired-end-Lib1/s_20_3_1_sequence.fq | 1200792298 | Y |  |  |  |  |
| Paired-end-Lib1/s_20_2_single_sequence.fq | 98852589 | Y | Y | Y |  |  |
| Paired-end-Lib1/s_20_2_2_sequence.fq | 1236163032 | Y | Y | Y |  | Y |
| Paired-end-Lib1/s_20_2_1_sequence.fq | 1192179990 | Y |  |  |  |  |
| Paired-end-Lib1/s_20_1_single_sequence.fq | 50064151 | Y |  |  |  |  |
| Paired-end-Lib1/s_20_1_2_sequence.fq | 1179134310 | Y | Y |  |  |  |
| Paired-end-Lib1/s_20_1_1_sequence.fq | 1153771208 | Y |  |  |  |  |
| Paired-end-Lib1/s_15_8_single_sequence.fq | 41030512 | Y | Y |  |  |  |
| Paired-end-Lib1/s_15_8_2_sequence.fq | 808514899 | Y | Y | Y | Y |  |
| Paired-end-Lib1/s_15_8_1_sequence.fq | 693952883 | Y |  |  |  |  |
| Paired-end-Lib1/s_112_1_2_sequence.fq | 2964338890 | Y | Y | Y | Y |  |
| Paired-end-Lib1/s_112_1_1_sequence.fq | 2964338890 | Y |  |  |  |  |
| Mate_pair/s_88_7_single_sequence.fq | 2306807 | Y |  |  |  |  |
| Mate_pair/s_88_7_2_sequence.fq | 147234557 | Y |  |  |  |  |
| Mate_pair/s_88_7_1_sequence.fq | 161236099 | Y |  |  |  |  |
| Mate_pair/s_87_7_single_sequence.fq | 1483921 | Y |  |  |  |  |
| Mate_pair/s_87_7_2_sequence.fq | 25045272 | Y |  |  |  |  |
| Mate_pair/s_87_7_1_sequence.fq | 26296226 | Y |  |  |  |  |
| Mate_pair/s_20_8_single_sequence.fq | 5375009 | Y |  |  |  |  |
| Mate_pair/s_20_8_2_sequence.fq | 101319138 | Y |  |  |  |  |
| Mate_pair/s_20_8_1_sequence.fq | 102906569 | Y |  |  |  |  |
